# Domain-Scan: Combinatorial Sero-Diagnosis of Infectious Diseases Using Machine Learning

**DOI:** 10.3389/fimmu.2020.619896

**Published:** 2021-02-10

**Authors:** Smadar Hada-Neeman, Yael Weiss-Ottolenghi, Naama Wagner, Oren Avram, Haim Ashkenazy, Yaakov Maor, Ella H. Sklan, Dmitry Shcherbakov, Tal Pupko, Jonathan M. Gershoni

**Affiliations:** ^1^ The Shmunis School of Biomedicine and Cancer Research, George S. Wise Faculty of Life Sciences, Tel Aviv University, Tel Aviv, Israel; ^2^ Max Planck Institute for Developmental Biology, Max Planck Society (MPG), Tübingen, Germany; ^3^ Institute of Gastroenterology and Hepatology, Kaplan Medical Center, Rehovot, Israel; ^4^ Department of Clinical Microbiology and Immunology, Sackler School of Medicine, Tel Aviv University, Tel Aviv, Israel; ^5^ Russian-American Anti-Cancer Center, Altai State University, Barnaul, Russia

**Keywords:** sero-diagnostics, DNA barcodes, next-generation sequencing, machine learning, phage-display

## Abstract

The presence of pathogen-specific antibodies in an individual’s blood-sample is used as an indication of previous exposure and infection to that specific pathogen (e.g., virus or bacterium). Measurement of the diagnostic antibodies is routinely achieved using solid phase immuno-assays such as ELISA tests and western blots. Here, we describe a sero-diagnostic approach based on phage-display of epitope arrays we term “Domain-Scan”. We harness Next-generation sequencing (NGS) to measure the serum binding to dozens of epitopes derived from HIV-1 and HCV simultaneously. The distinction of healthy individuals from those infected with either HIV-1 or HCV, is modeled as a machine-learning classification problem, in which each determinant (“domain”) is considered as a feature, and its NGS read-out provides values that correspond to the level of determinant-specific antibodies in the sample. We show that following training of a machine-learning model on labeled examples, we can very accurately classify unlabeled samples and pinpoint the domains that contribute most to the classification. Our experimental/computational Domain-Scan approach is general and can be adapted to other pathogens as long as sufficient training samples are provided.

## Introduction

B-cells respond to infection by producing pathogen-specific antibodies that bind and neutralize infectious agents (1, 2). In addition to clearance of pathogens, memory B-cells are deposited as an immunological archive to be called upon in the event that a specific pathogen is re-encountered. Hence, the repertoire of antibodies in our serum contains medically relevant information regarding past and present interactions with pathogens, that is revealed through various serological immuno-assays (3, 4).

The “AIDS” test for example, measures the presence of HIV-specific antibodies in a given serum sample being examined (5). The presence of HIV-specific antibodies indicates that the subject encountered the virus in the past. Ideally, such immunoassays should be 100% specific, that is, never mis-classifying a healthy individual and 100% sensitive, i.e., never missing a *bona fide* infected person. Unfortunately, specificity and sensitivity are never perfect. In order to increase accuracy, several repeats of a test are performed, often using more than one methodology. For example, Enzyme-Linked Immunosorbent Assay (ELISA) tests are routinely run in duplicates and triplicates. However, further confirmation of positive ELISA test results is possible through western blot analysis (6, 7). Greater diagnostic confidence by western blot is gained when positive signals can be associated with multiple viral peptides. This effectively discriminates between fortuitous cross-reactive antibody binding to a single viral antigen, compared to multiple signals associated with different viral antigens, resolved by electrophoresis. The latter reflects multiple B-cell encounters with the pathogen that consequently produced a variety of antibodies against a spectrum of virus epitopes.

**Figure 1:**
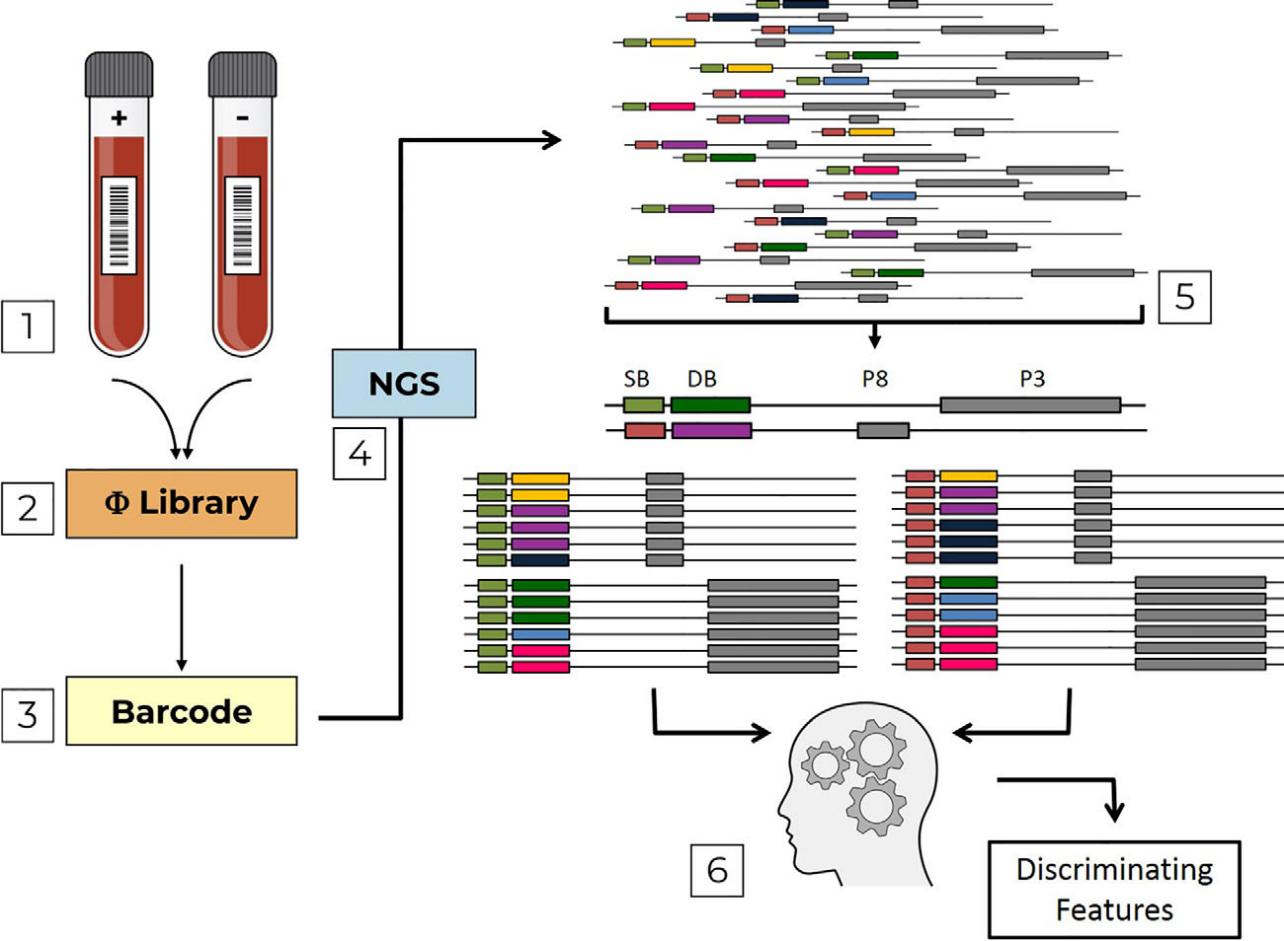
Pipeline flow chart. The pipeline is composed of sequential steps; (1) Serum samples, which are either positive (+) or negative (-) with respect to a specific infection are used to screen Domain-Scan phage-displayed libraries (2) During PCR sample preparation, sample barcodes are incorporated into the sequence affinity selected phages (3) that are then sent to NGS (4). Erroneous DNA reads are filtered out. Then, the remaining reads are parsed (5) first by sample barcodes (SB, green and orange) and then by domain barcodes (DB, yellow, violet, blue, dark-green, light blue and pink). The two libraries (PVIII and PIII) are analyzed by a machine-learning algorithm (6) to identify discriminating features.

Previously, we proposed a novel method for sero-diagnosis in which individual epitope arrays are used as bait for the detection of pathogen-specific antibodies (8). This method of “Combinatorial Diagnostics” has the potential for scale-up, which could enable the multiplex testing for numerous pathogens in a single sample. Here we report further development of “Combinatorial Diagnostics”, combining biopanning of antigen-based filamentous phage-display libraries with Next-generation sequencing (NGS), namely “Deep Panning” (9). Moreover, we implement a computational pipeline to analyze our data and construct machine-learning models in order to discriminate between different groups of sera (**Figure 1**).

## Materials and Methods

The main purpose of this study is to provide a multiplex and potentially high throughput platform for the sero-diagnosis of multiple infectious diseases. This is based on scoring serum antibody responses to arrays of pathogen defined peptides (15-50 amino acids), “Domains”. In order to test multiple serum samples against multiple pathogen peptide arrays simultaneously in a single sample, NGS data have been analyzed *via* a computational pipeline implementing machine learning.

### Construction of the “fth1-BC” Vector

**Figure 2:**
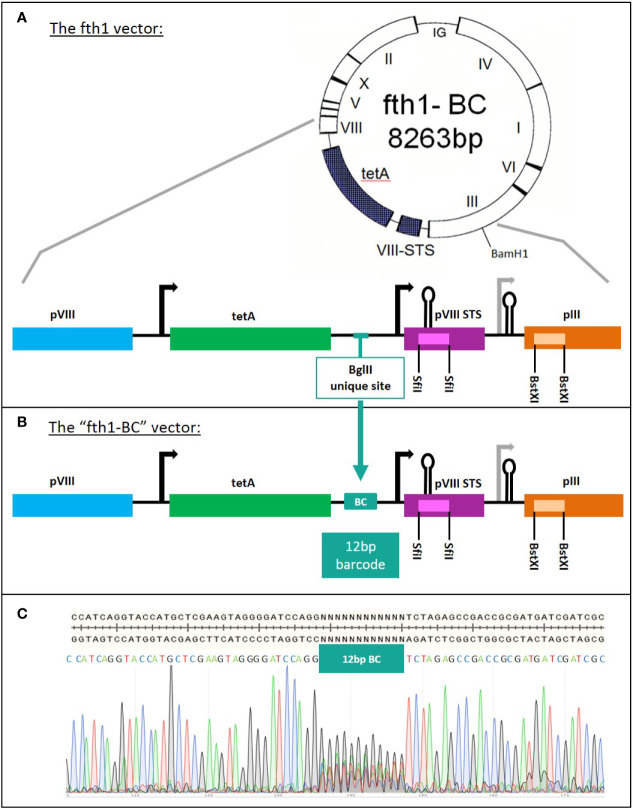
The construction of the “fth1-BC” phage-display vector. **(A)** fth1 vector: the fth1 vector has two cloning cassettes, in recombinant protein VIII (SfiI sites, purple) and in protein III (BstXI sites, orange). **(B)** The “fth1-BC” vector has been modified such that recombinant protein VIII and protein III genes are preceded by a barcode (BC) coding region. The barcode region codes for a total of ~1.6x10^7^ unique 12-bp DNA barcodes. In **(C)**, the “fth1-BC” sequence and the Sanger sequencing-chromatogram illustrate the randomness of the barcode sequence.

The fth1 filamentous bacteriophage vector (10) was modified to incorporate a library of barcodes, thus producing the “fth1-BC” vector (**Figure 2**). Oligonucleotides were designed to insert 12 random-base barcodes (N = A/C/G/T) into an untranslated region of the fth1 phage:

Sense oligonucleotide: 5’-TAGGGGATCCAGGNNNNNNNNNNNNTCTAGAGCCGACCG-3’

The anti-sense oligonucleotide (5’- CGGTCGGCTCTAGA-3’) was complementary to the 14 bases at the 3’ end of the sense oligonucleotide (underlined). The two oligonucleotides were annealed and filled in with Klenow DNA polymerase large fragment (NEB cat# M0210L, MA). The insert containing the 12 random-bases was cloned into *Bgl*II-digested fth1 vector, using a Gibson assembly reaction (11) (40 bases corresponding to the fth1 sequences were added to the ends of the construct *via* PCR amplification). The cloned vectors were transformed into *E. coli* MC1061 by electroporation. The transformed cells were cultured in 500 ml of LB medium with tetracycline (20 μg/ml). The culture was grown overnight at 37°C with shaking at 225 rpm. The culture was then centrifuged at 8,000 rpm, for 20 min. The supernatant was discarded, and the pellet was used for plasmid DNA extraction (NucleoBond^®^ Xtra Maxi Plus, MN, Düren, Germany).

### Construction of Domain-Scan Libraries

All the Domain-Scan libraries were generated and expressed using the “fth1-BC” barcoded expression vector. Generally, unless indicated otherwise, for each target antigen, corresponding DNA primers were designed to produce serial overlapping segments (15 aa, 20 aa, or 50 aa in length) starting at the first codon of each gene and shifting by 15 or 30 bases (5 aa or 10 aa) towards the 3’ end of the gene. The size of each domain in every library was validated in agarose gel, and each insert was cloned into the digested and purified “fth1-BC” vector. The cloned vectors were used to transform DH5alphaF- competent bacteria. Clones were isolated and sequenced by standard Sanger sequencing in order to confirm the correctness of each cloned domain-peptide sequence, and to determine its corresponding barcode.

A total of six Domain-Scan libraries representing antigens derived from HCV and HIV-1 were cloned and expressed in fth1 Protein III and Protein VIII (the amino acid sequences are detailed in **Table S1**). Two cloning procedures were used for the Protein VIII libraries:

### HIV-1 p24

Oligonucleotides corresponding to peptides 15 aa long and shifted every 5 aa corresponding to the HIV-1 p24 antigen, were designed (sense and anti-sense) such that, upon annealing, 3’-overhangs corresponding to the overhangs generated after cleavage of the 5’ and 3’ *Sfi*I sites of recombinant Protein VIII were produced. The dsDNA products were directly ligated into the *Sfi*I-digested “fth1-BC” by T4 DNA ligase.

### HCV-CORE, HCV-E2 Domain-Scans, and Selected Peptides Derived From HCV-E2 and NS3

For the production of Domain-Scans, sets of oligonucleotides were designed to correspond to overlapping peptides of 15 aa shifted every 5 aa, and 20 aa shifted every 5 aa for HCV-CORE and HCV-E2 antigens, respectively. The oligonucleotides corresponding to each segment, were annealed (sense and anti-sense oligos were designed to have complementary 3’ ends), and the 5’ ssDNA aspects of the constructs were filled in with Klenow DNA polymerase. The products were ligated into *Sfi*I-digested “fth1-BC” vector *via* a Gibson assembly. The same general scheme was used for the selected E2 and NS3 peptides (see **Table S2**).

### Construction of Three Domain-Scans (HCV-NS3, HCV-NS5, and HIV-1-gp160) in Protein III

Oligonucleotides corresponding to overlapping 50 aa peptides with 10 aa shifts were prepared and cloned into Protein III. For this, gBlock DNA templates corresponding to HCV-NS3 and HCV-NS5 antigens of HCV genotype 1b were purchased from IDT (Integrated DNA Technologies, IA, USA). The template used for the production of the HIV-1 gp160-ConS peptides were kindly provided by Dr. Barton Haynes (12). Oligonucleotide primer-pairs (sense and anti-sense) were designed to enable PCR amplification of each of the specific overlapping and shifted domains, using the DNA templates described above. The primers included 5’ 20-base sequences corresponding to the *protein III* gene sequence flanking either side of the *BstX*I sites of the “fth1-BC” vector. Each domain was cloned, individually, into *BstX*I-digested “fth1-BC” vector using a Gibson assembly reaction. Each clone was sequenced to validate the sequence for correctness and to identify its corresponding 12-bp domain-specific barcode. Similarly, two selected peptides of 68 aa and 76 aa derived from the NS3 antigen were designed and cloned into the *BstX*I sites using the same general procedure (see **Table S2**).

Once the DNA clones, corresponding to the peptides derived from the six antigens used in this study were confirmed and their barcodes determined, they were consolidated respectively, to produce six individual DNA mixtures (Protein VIII: HCV-CORE, HCV-E2, HCV-NS3, HIV-1-p24, and Protein III: HCV-NS3, HCV-NS5, and HIV-1-gp160). For this, 150 ng DNA aliquots from each domain were combined to produce the six separate DNA mixtures. Next, the three DNA mixtures of Protein VIII-expression libraries were combined into one library (consolidated Protein VIII Library). Similarly, the DNA mixtures of the three Protein III-expression libraries were combined as well (consolidated Protein III Library). The consolidated Protein VIII and Protein III DNA mixtures were used separately to transform competent DH5alphaF-. Cultures were grown, and supernatants were collected. Bacteriophages were precipitated with polyethylene glycol (PEG-NaCl) overnight at 4°C, centrifuged at 8,000 rpm to harvest the bacteriophages, and re-suspended in Tris-buffered saline (TBS).

### Polyclonal Sera

Polyclonal serum-samples were collected from three sources: The Israeli National Blood Bank (Magen David Adom, Tel Hashomer), Chaim Sheba Medical Center and Altai State University. A total of 85 different samples were analyzed in this study, representing three biological conditions: 15 HIV-1 positive, 40 HCV positive, and 30 healthy individuals. All sera were collected under informed consent and approved by the Institutional Review Boards. All samples were stored at -20°C.

### Biopanning of Domain-Scan Phage-Display Libraries

For biopanning, 10 µl (approximately 1x10^10^ phages) of the Protein VIII or Protein III Domain-Scan libraries were mixed with 1 µl of serum sample and 1x10^10^ *wt*-fth1 phages (“carrier” phages were added with the intent to reduce non-specific signals), in TBS containing 3% BSA (completed to 100 µl) and incubated for 1 h on a rotating mixer at room temperature. Next, 50 µl of Protein-G coated magnetic beads (Invitrogen, Dynabeads^TM^ Protein G) were added, and the mix was incubated for an additional 30 min on a rotating mixer at room temperature. The vials were then placed on a magnetic stand (Promega, MagneSphere^®^ Technology Magnetic Separation Stands) for 2 min to collect the beads, and the supernatants were discarded. Subsequently, the beads were washed three times with 200 µl ice-cold Tris-buffered saline containing 0.5% Tween-20 detergent (TBST), re-suspended in 100 µl TBST, and transferred to a new vial. The vials were then placed on the magnetic stand, and the supernatants were discarded. Bound phages were eluted with 105 μl of elution buffer (0.1 M HCl adjusted to pH 2.2 with glycine, 1 mg/ml BSA) for 10 min at room temperature. The eluate was collected and neutralized with 19 μl of neutralizing buffer (1 M Tris-HCl pH 9.1). Each serum tested was used to screen the Domain-Scan libraries in triplicate, thus generating three independent samples sent to NGS. In order to multiplex dozens of samples together on a single NGS chip, an 8-bp sample-indexing barcode was introduced by PCR during sample preparation (see below). Hence, each serum sample was analyzed in triplicate, thus corresponding to three sample-indexing barcodes.

### Sample Preparation for Illumina NGS Sequencing

PCR amplification of the Domain-defining barcodes was preformed directly on the eluted phages with no further DNA purifications. PCR reactions were conducted using the following primers introducing the Illumina Adaptor A and Adaptor B sequences, respectively:

“Forward”

5’-AATGATACGGCGACCACCGAGATCTACACTCTTTCCCTACACGACGCTCTTCCGATCT

xxxxxxxxAAGTAGGGGATCCAGG-3’

(“xxxxxxxx” represents the unique 8-nt sample-indexing barcode, see above).

“Reverse”

5’-CAAGCAGAAGACGGCATACGAGCTCTTCCGATCTATCGCGGTCGGCTCTAGA-3’.

For each sample, 1 µl of the eluted phages, was mixed with 12.5 µl high-fidelity DNA polymerase (Invitrogen, Platinum^TM^ SuperFi™ PCR Master Mix), 10 pmol of “Forward” and “Reverse” primers, and ultra-pure H_2_O to a total reaction volume of 25 µl. The thermal profile was:

1. 98°C 2 min
2. 98°C 10 s
3. 60°C 10 s
4. 72°C 10 s
  Go back to step 2 x25
5. 72°C 5 min

PCR product size was validated on 2% agarose gels, purified by Agencourt AMPure XP–PCR Purification (Beckman Coulter) and measured for concentration using a Qubit fluorometer. The samples were then diluted to 10 nM, combined and sent for Illumina NGS sequencing. Hence, three “samples”, each tagged with an 8-bp indexing-barcode, were produced for every serum that was analyzed. NGS output DNA reads contained a sample-indexing barcode followed by a Domain-defining barcode, a total of 54 bp fragment, flanked by the Illumina Adaptor-sequences (the total length of the PCR fragment was 152 bp).

### Next-Generation Sequencing (NGS)

NGS was performed at the Weizmann Crown Institute for Genomics. 11 pM of samples were used for clustering PCR followed by sequencing of 1x75 bp using an Illumina NextSeq500. The lane was spiked with 20% PhiX DNA. Approximately 100 samples were run on a single lane and a total of six runs were conducted in this study. The 85 serum samples (see above) were screened against the six Domain-Scan libraries representing 339 domains (**Table 1**). A total of 655,017,893 reads were generated and used as input for the computational analysis.

**Table 1:** Domain-Scan libraries.

	HIV-1		HCV			
**Antigen**	p24	gp160*	CORE	E2	NS3	NS5
**Length of peptide**	15aa	50aa	15aa	20aa	50aa	50aa
**No. of peptide**	41	83	27	75	81	41
**Shift**	5aa	10aa	5aa	5aa	10aa	10aa
**Displayed on**	pVIII	pIII	pVIII	pVIII	pIII	pIII
**Genotype**	HIV-1	HIV-1	1b	1b	1b	1b

For each antigen, we designed a library of overlapping peptides of various lengths and starting points. Peptides of 15 aa and 20 aa in length were cloned into pVIII, and peptides of 50 aa in length were cloned into pIII. Each of the HCV-NS3 and the HCV-E2 Domain-Scan libraries contain a few additional selected peptides (**Table S2**). *9 domains were not used in the analysis due to technical problems (i.e., only 339 domains were used, see **Table S2**).

### Computational Analysis Input

The input to the machine-learning classifier was a list of sequence counts for each domain in each sample (the list of HIV-1 and HCV domains is given in **Table S2**, and the complete corresponding raw data are provided in **Table S3**).

### Data Pre-Processing

First, samples for which there were less than 100,000 total counts were filtered out. Three and two such samples were filtered in the Protein III and Protein VIII libraries, respectively. For each of the remaining samples, the counts for irrelevant domains were used for normalization. Specifically, when classifying HIV-1 against healthy individuals, the counts for the HCV domains were used for normalization, while when classifying HCV against healthy individuals, the HIV-1 domains’ counts were used for normalization. Normalization was done by dividing each count by the total counts for the irrelevant domains. The rationale for using irrelevant domains for normalization is that the irrelevant domains measure non-specific binding. Thus, this normalization provides an estimate of the fold enrichment of domains of interest (either HIV-1 or HCV) relative to “noise”. For example, when the sample is from an HIV-1 positive sample, the normalized ratio for an HIV-1 domain should be high. In contrast, for an HIV-1 negative sample, the ratio should be substantially lower.

An average over the normalized counts of the triplicates of each domain, per individual, was computed to form a single sample per individual, i.e., after these steps, we obtained 85 samples, corresponding to the number of individuals. The data for each domain before and after processing are given in **Tables S3** and **S4**, respectively.

### Machine-Learning Classification, Training, and Test Data

We considered the problem of identifying whether a patient is infected with HIV-1 or not as a machine-learning binary classification problem (and similarly for HCV). For each sample, the input to the machine-learning algorithm was sequence counts of each domain (HIV-1 and HCV domain names are given in **Table S2**, and the complete corresponding raw data are provided in **Table S3**). We used the labeled cases described above to train machine-learning classifiers. These training data included 80% of both the infected and healthy individuals, while 20% were kept aside for testing, as described in a sequel. The machine-learning classifiers were trained to optimally separate the infected individuals from the healthy ones (based on the domain counts as features). To test the accuracy of the trained classifiers, 20% of all individuals (9 and 14 for HIV-1 and HCV, respectively) were randomly selected to compile a test dataset. Specifically, these 20% individuals were selected so that the ratio between infected and healthy individuals was identical in the training and test data (i.e., stratified sampling). The test data, for which we actually know the “true label”, were kept aside during the training step. The training and test data are provided in **Table S5**.

### Machine-Learning Classification, Algorithms

The following classifiers were tested: Naïve Bayes (NB), Support Vector Machine (SVM), Logistic regression (LR), Linear-Discriminant Analysis (LDA), Random Forest (RF), and k-Nearest Neighbors (KNN). We used the Python implementation of these algorithms, available in the “Scikit-learn” package (13).

### Machine-Learning Training and Feature Selection

For some domains, the median value of the healthy individuals was found higher than the median of infected individuals, indicating that these domains bind serum non-specifically. Thus, based on the training set, these domains were excluded from both the training and the testing data. To test the accuracy of each classifier on the training data, we used a repeated five-fold cross-validation procedure. Specifically, we divided the training data to five disjoint folds, each comprising 20% of the training data. Here too, we applied stratified sampling to form the folds. Each classifier was trained on four folds (comprising 80% of the training data) and its performance was evaluated on the remaining fifth fold using the AUC (area under the ROC curve) score. This process was repeated five times, each time with a different fold used for performance evaluation. This process of dividing to five random folds was repeated 50 times and the average AUC was measured over the 250 folds used for validation.

The above repeated cross-validation procedure on the training data was used both for parameter tuning and for additional feature selection, performed for each classifier. Parameter tuning was used both to improve the classifier performance and to avoid overfitting, i.e., accurate classification of the training data and poor classification of the validation data. Model regularization was introduced by limiting the number of trees, the tree depth, and the number of samples in the nodes of the tree in the Random Forest classifier, and by introducing the Lasso and Ridge Regression in the logistic regression classifier. The following feature-selection methods were considered: recursive feature elimination and select from model, as implemented in Scikit-learn (13). The set of features providing the highest AUC following these two feature-selection methods was chosen. After this procedure, we selected the classifier with its chosen parameters and its associated set of features that provided the highest AUC on the training data and evaluated its performance on the test data. Of note, the feature selection procedure additionally provides a feature importance score for each feature, reflecting its contribution to the classification accuracy.

### Sequencing Noise Analysis

To examine a potential effect of sequencing noise produced by the NGS on our analysis we applied two complementary approaches: (i) We tested how stable our results are. To do so, instead of averaging the triplicates we randomly selected a single sample from each triplicate. We then repeated the entire machine-learning pipeline as described above, this time using the single samples instead of the average over triplicates; (ii) We tried to test how sequencing coverage affects the obtained results. To do so, we followed a down-sampling approach, in which we randomly sampled a subset of the reads. Specifically, we sampled 50%, 60%, 70%, 80%, and 90% of the reads. We repeated the entire machine-learning pipeline for each such sampling.

## Results

Combinatorial diagnostics is based on individually measuring the binding of serum-antibodies to members of a panel of pathogen-defining markers. In this study, we applied Deep-Panning analysis of pathogen-defining Domain-Scan peptides, displayed on filamentous bacteriophages in order to develop a high throughput and multiplex serum diagnostic platform.

Initially, we used Protein VIII to express 105 short peptides derived from the CORE, E2 and NS3 antigens of HCV and 41 peptides representing p24 of HIV-1. Subsequently, in order to expand the range of diagnostic antigens and potentially include conformational targets, we added 205 “domains” as Protein III fusions representing HCV NS3 and NS5 as well as HIV-1 gp160. Ultimately, a total of 339 peptides, representing “domains” derived from six different antigens of HIV-1 and HCV, were analyzed.

### Construction of the “fth1-BC” Vector

The Domain-Scan libraries were prepared using a novel fth1-BC vector system. In order to obtain an optimized and uniform read-out using NGS, we modified the fth1 vector by inserting a barcode-library of 12 random bases into an intergenic region of the vector. Each cloned domain was thus associated with a unique 12-bp barcode (see *Materials and Methods* and panels A and B in **Figure 2**). As a result, all peptide domains, irrespective of their composition, length, or fusion partner (Protein VIII or Protein III) were scored by NGS with equal read efficiency.

An aliquot of the modified fth1 barcode (BC) vector was sequenced to confirm the expected high variability of the barcode region (**Figure 2C**). Furthermore, the barcodes of twenty randomly selected clones were sequenced and found to be different from each other. The vast complexity of barcodes was further shown by reading an aliquot of the library by NGS.

This “fth1-BC” vector was used as a universal platform for producing the Domain-Scan libraries in either the *protein III* gene (using the *BstX*I sites) or *protein VIII* gene (using the *Sfi*I sites). As a result, each cloned peptide could be associated with a unique barcode. The sample preparation for Illumina NGS was performed by PCR, employing sense and anti-sense primers containing the Adaptor A and B sequences, respectively (see Materials and Methods). Moreover, a sample indexing-barcode was introduced just prior to the domain-defining barcode. The PCR product (152-bp) was “ready to use”, generating reads of uniform size containing first the sample index barcode (8-bp) followed by the domain barcode (12-bp), a total uniform read length of 54-bp irrespective of the size of the domain or whether it was displayed on Protein III or Protein VIII (a general scheme of the experimental procedure is given in **Figure 1**).

Panning of phage libraries can sometimes generate non-specific binding due to the “stickiness” of peptides. In order to reduce this “background noise”, the Domain-Scan libraries were mixed with carrier “inert” phages, fth1-phages devoid of any insert. In addition, we designed the sequences flanking the 12-bp DNA barcodes to be unique sequences (i.e., absent in the fth1 phage), and as a result, the PCR primers used for NGS sample preparation did not generate signals from the fth1 vector DNA. Thus, the dilution of the Domain-Scan phage-display libraries with carrier phages, led to decreased background noise, without introducing reads from fth1 carrier phages.

### Construction of Domain-Scan Libraries

**Figure 3:**
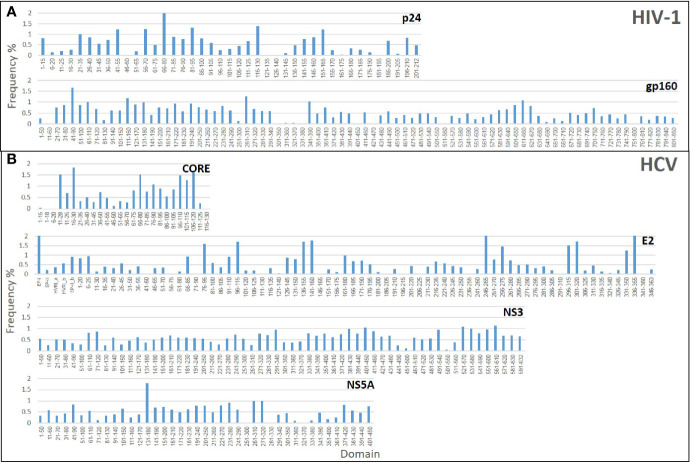
Baseline analysis of the Domain-Scan libraries: The histograms represent the frequency distributions of the various domains that constitute each library. **(A)** HIV-1 libraries **(B)** HCV libraries.

Initially, three Domain-Scan libraries were prepared by cloning oligonucleotides corresponding to short peptides into the *Sfi*I cloning sites of the recombinant *protein VIII* gene of the fth1-BC vector; (i) 41 overlapping domains of the p24 antigen of HIV-1, (ii) 27 domains from the N-terminal aspect of HCV-CORE antigen (residues 1-135), and (iii) 75 domains representing HCV-E2 antigen. In addition, phages expressing selected non-overlapping peptides derived from the HCV-NS3 antigen were also prepared (see **Table S2**). In order to expand the libraries and include potential conformational diagnostic epitopes, three additional Domain-Scan libraries were produced displaying longer peptides. For this, the *BstX*I cloning sites of the recombinant *protein* III gene of the fth1-BC vector were employed as Protein III can incorporate relatively large peptide inserts (14). The libraries were as follows: (i) 83 domains representing HIV-1 ConS gp160 antigen, (ii) 81 domains representing HCV NS3 antigen, and (iii) 41 domains representing HCV NS5A antigen (**Table 1**). A total of 348 clones were produced, each associated with a unique 12-bp domain-defining DNA barcode. Once all domains and barcodes were confirmed for each of the antigens, the Protein III clones and the Protein VIII clones were combined to yield two individual DNA mixtures, which were used separately to transform *E. coli* cells for phage production. **Figure 3** illustrates the “landscape baselines” of the domains for each of the antigens used as was measured by NGS performed directly on the naïve unscreened libraries.

**Deep-Panning of the Domain-Scan Libraries:** Serum samples representing three biological conditions were used to biopan the consolidated combined phage-displayed Domain-Scan libraries; sera from: 40 HCV infected, 15 HIV-1 infected, and 30 naïve healthy individuals (a total of 85 serum samples). The serum samples were screened in triplicate against each of the two phage-display Domain-Scan libraries (for details, see Materials and Methods). For each serum sample, an additional “sample-defining” 8-bp DNA barcode was introduced during the sample preparation for NGS (**Figure 1**).

### Computational Analysis of the Domain Scan

The 85 serum samples were screened in triplicate and the affinity-selected phages were harvested, processed, and their DNA was sent to NGS, yielding a total of 655,017,893 reads (**Table 2**). The next step was to analyze the extensive amount of data obtained from deep-panning experiments, which reflect affinity-selected domains from multiple serum samples, in order to draw conclusions with respect to the affinity-selected domains and their application as diagnostic markers.

**Table 2:** Deep-Panning of HCV and HIV-1 Domain-Scan libraries.

	HCV	HIV-1	Naïve
**Number of serum samples**	40	15	30
**Reads: pIII-fusions**	151,989,575	54,974,260	146,232,618
**Reads: pVIII-fusions**	139,566,764	49,070,090	113,184,586
**Total reads**	291,556,339	104,044,350	259,417,204

85 sera derived from three groups: HCV infected individuals, HIV-1 infected individuals, and healthy individuals (naïve) were screened as three independent repeats, against the two consolidated Domain-Scan libraries (pIII-fusions and pVIII-fusions). The DNA of the affinity-selected phages was sequenced, and the number of reads for each group and total reads are given.

We developed a computational pipeline, providing a high-throughput, and multiplex platform for sero-diagnosis of infectious diseases (**Figure 1**). The pipeline was comprised of four sequential steps: (i) Quality control of the sequences, in which aberrant reads were discarded; (ii) Parsing the sequences, first by sample-barcodes and then by domain-defining barcodes; (iii) Calculation of an “enrichment score” and removal of irrelevant domains; and (iv) Machine-learning classification aimed to discriminate healthy from infected individuals and quantify the contribution of each disease-related domain to the classification performance.

Steps (i) and (ii) are described in detail in the Materials and Methods. Step (iii) is the calculation of an “enrichment score” for each domain and determination of the utility of a domain as a discriminating diagnostic marker. This was conducted in a number of steps as described herewith: For each serum sample, a set of disease-related domains were identified and compared against “irrelevant domains”. As a case in point, the Protein VIII domains derived from the CORE, E2 and NS3 antigens are regarded as “disease-related domains” when analyzing HCV. Consequently, the p24 HIV-1 derived domains were taken as the “irrelevant domains” for comparison. For this, the reads of the p24 HIV-1 domains were summed and used as a reference “normalization-constant”. Then, the observed reads for each HCV-domain were divided by the normalization-constant, resulting in a “domain-enrichment score”. For the naïve healthy sera, enrichment scores were calculated in the same manner (i.e., when HCV sera were analyzed both the HCV positive and the healthy samples were divided by the sum of HIV-1 domains, and when the HIV-1 sera were analyzed, both the HIV-1 positive and the healthy individuals were divided by the sum of the HCV domains). Next, the irrelevant domains were removed from the analysis (**Table S4**). Finally, the enrichment scores of the disease-related domains were used for the subsequent machine-learning classification.

### Machine-Learning Classification

First, the data were randomly divided into two distinct sets: 80% of the data were used for training and 20% of the data were left aside in order to be used as a test set to assess the ability of the platform to diagnose the clinical status of un-seen sera. After training the classifiers on the training data as described in the methods, the classifier was fitted to the training set and the AUC was calculated both for the training data and for the test data. In total, six classifiers were evaluated, as described in the methods.

We first analyzed only the data obtained from the Protein VIII Domain-Scan library containing; CORE (26 domains), E2 (75 domains), and NS3 (17 domains) for HCV, and p24 (41 domains) for HIV-1. A total of 69 domains remained after discarding the irrelevant domains: 22, 43, 3, and 1 for HCV-CORE, HCV-E2, HCV-NS3, and HIV-1-p24, respectively.

Regarding the HCV classification, the Random Forest classifier outperformed all other classifiers with a perfect classification (AUC of 1.0) both on the training set and on the test data. In addition to AUC, the results of the classification can also be described in a confusion matrix, which provides the frequencies of true positives (correctly identified infected individuals), false positives (healthy individuals misidentified as infected), true negatives (correctly identified healthy individuals), and false negatives (infected individuals misidentified as healthy). The HCV training set included 32 infected and 24 healthy serum samples, while the test set included eight HCV-infected and six non-infected serum samples and the accuracy of the HCV classification using Random Forest was 100% both on the training and the test sets.

Regarding HIV-1 classification (based on the Protein VIII Domain-Scan library), only one domain remained after discarding the domains for which the median of the healthy individuals was higher than that of infected individuals (see *Materials and Methods*). This domain had a median of 0 both in the healthy and infected individuals and consequently, no further analysis could be done on the data. It suggests that p24 domains alone provide only poor sero-diagnostic power for HIV-1.

These results led us to construct additional domain scans expressed on Protein III, assuming that by using additional larger peptide segments of viral antigens (50aa) we might gain conformational epitopes as well. We added Domain-Scans for both HIV-1 and HCV, anticipating that HIV-1 classification will now become feasible. We expected the already perfect HCV classification to remain the same. The domains added in the Protein III Domain-Scan consolidated library included NS3 and NS5A for the HCV (61 and 40 domains, respectively) and gp160 (79 domains) for HIV-1. A total of 71 domains remained in this library after discarding the irrelevant domains (5 for HCV-NS3, 25 for HCV-NS5A, and 41 for HIV-1 gp160).

Regarding HCV classification, Random Forest showed the best performance with an AUC of 0.999 on the training and 0.875 on the test. Combining the domains of both the Protein VIII and Protein III libraries resulted with a perfect AUC of 1.0 both on the training and the test sets (with Random Forest as the best classifier). Thus, the combined analysis maintained the perfect AUC score achieved using the Protein VIII library domains alone, both for the training and the test datasets. The accuracy remained perfect as well.

The inclusion of 50-residue gp160 domains enabled accurate HIV-1 classification. The best performing classifier was Random Forest, and the resulting AUC was perfect (AUC of 1.0) both for the training and test sets. The corresponding accuracy was also perfect.

**Figure 4:**
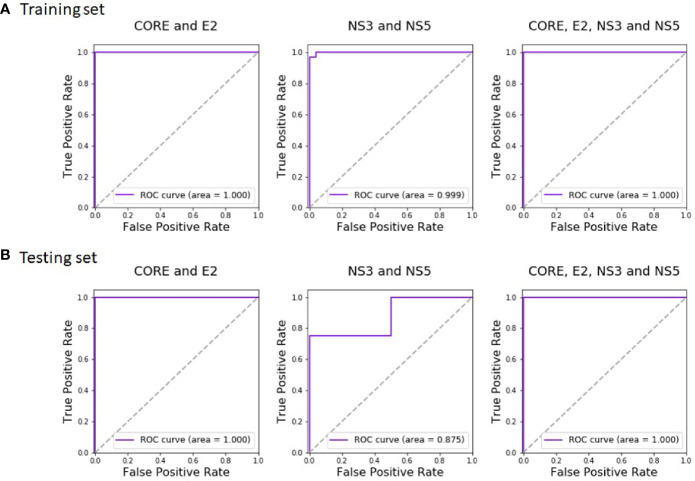
Random Forest ROC-curves of HCV training set and test set. The curves were plotted based on the **(A)** training set predictions and **(B)** test set predictions. The predictions were done after training the classifier on CORE and E2 domains (left), NS3 and NS5A domains (middle), and all four antigens (right).

The complete training and test results of all the classifiers, for the Protein VIII Domain-Scan library, for the Protein III Domain-Scan library, and for the combined analyses, are available in **Tables S6** and **S7**, for HCV and HIV-1, respectively. The corresponding Random Forest ROC curves of HCV are given in **Figure 4**. We additionally tested how robust the reported results are to sequencing noise (see Methods). Both relying on a single sample instead of triplicates and down sampling by 50% did not affect the results (i.e., a perfect classification for both HIV-1 and HCV), suggesting that sequence noise was not a major concern in this analysis.

**Figure 5:**
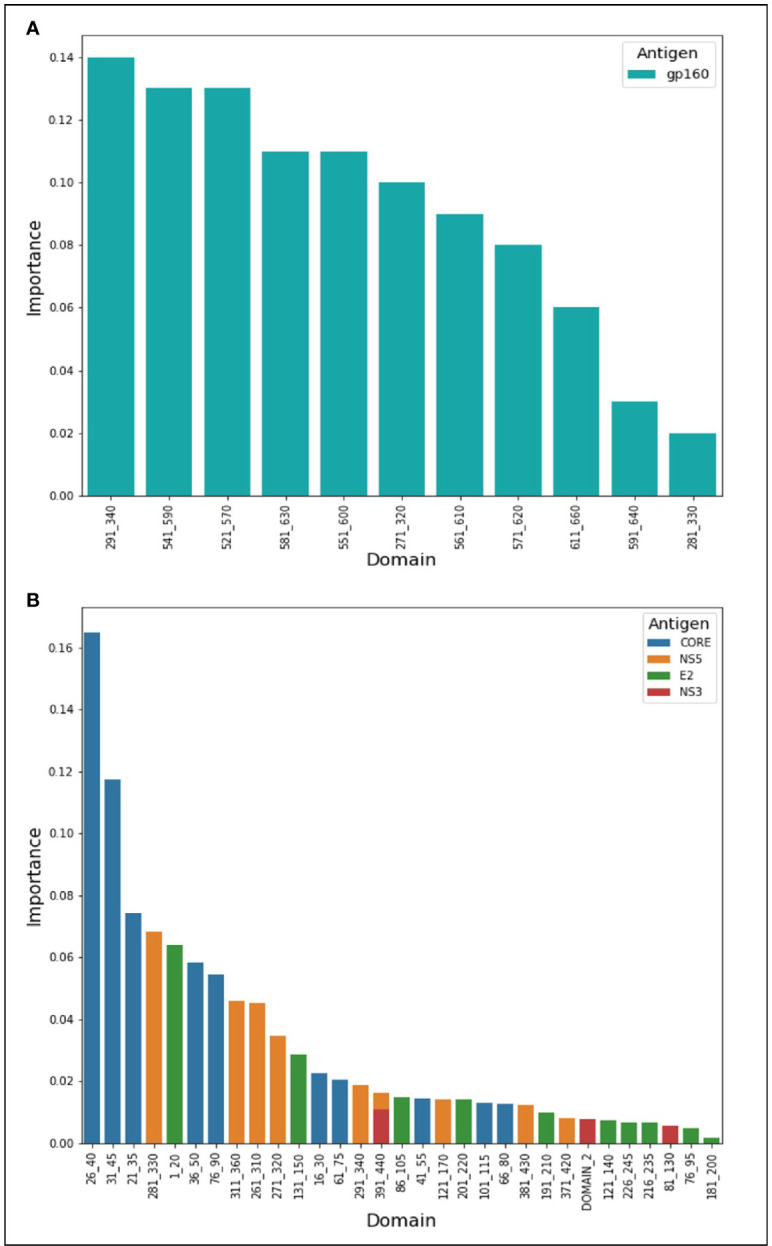
Features’ importance scores for HIV-1 and HCV domains. The selected domains were ordered in the x-axis from left to right according to their importance for Random Forest classification. The y-axis represents the importance score. In **(A)** are the domains of HIV-1 and in **(B)** are the HCV domains.

**Figure 6:**
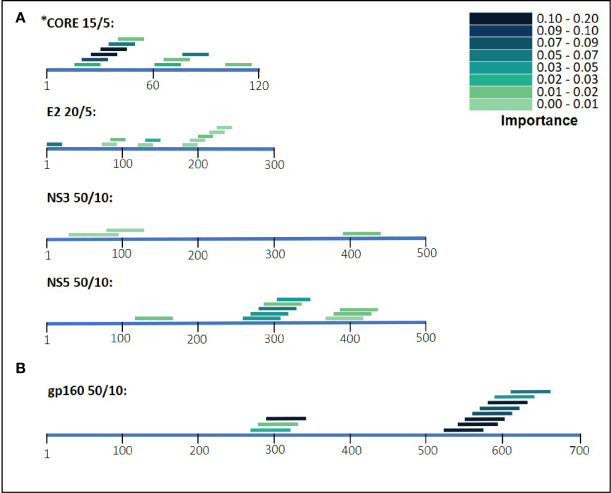
HIV-1 and HCV selected domains. The selected domains were ordered based on their position on the antigen’s amino acid sequence. The color density of the domains indicates the importance for Random Forest classification. In **(A)** are the domains of HCV and in **(B)** are the HIV-1 domains.

During the classification process, a feature-selection procedure was conducted in order to achieve better accuracy for the classification. Features (domains) that were identified as the most meaningful from our data were kept while the rest were discarded. The feature-selection procedure also provided a feature importance score for each feature, reflecting its contribution to the classification accuracy, the higher the score, the more important is the feature for classification. The most important features were CORE_26-40 for HCV and gp160_291-340 for HIV-1. Furthermore, the feature selection process selected domains from all the antigens (see feature importance bar plots in **Figure 5**). The distribution within the antigens of the most important features is shown in **Figure 6**. Seven clusters of overlapping features are apparent. The importance scores for these features are given in **Tables 3** and **4**.

**Table 3:** HIV-1 selected domains and the corresponding importance score.

HIV selected domains			
Antigen	position	Amino-acid sequence	Importance
gp160	271_320	SENITNNAKTIIVQLNESVEINCTRPNNNTRKSIRIGPGQAFYATGDIIG	0.1
	281_330	IIVQLNESVEINCTRPNNNTRKSIRIGPGQAFYATGDIIGDIRQAHCNIS	0.02
	291_340	INCTRPNNNTRKSIRIGPGQAFYATGDIIGDIRQAHCNISGTKWNKTLQQ	0.14
	521_570	TMGAASITLTVQARQLLSGIVQQQSNLLRAIEAQQHLLQLTVWGIKQLQA	0.13
	541_590	VQQQSNLLRAIEAQQHLLQLTVWGIKQLQARVLAVERYLKDQQLLGIWGC	0.13
	551_600	IEAQQHLLQLTVWGIKQLQARVLAVERYLKDQQLLGIWGCSGKLICTTTV	0.11
	561_610	TVWGIKQLQARVLAVERYLKDQQLLGIWGCSGKLICTTTVPWNSSWSNKS	0.09
	571_620	RVLAVERYLKDQQLLGIWGCSGKLICTTTVPWNSSWSNKSQDEIWDNMTW	0.08
	581_630	DQQLLGIWGCSGKLICTTTVPWNSSWSNKSQDEIWDNMTWMEWEREINNY	0.11
	591_640	SGKLICTTTVPWNSSWSNKSQDEIWDNMTWMEWEREINNYTDIIYSLIEE	0.03
	611_660	QDEIWDNMTWMEWEREINNYTDIIYSLIEESQNQQEKNEQELLALDKWAS	0.06

**Table 4:** HCV selected domains and the corresponding importance score.

HCV selected domains			
Antigen	Position	Amino-acid sequence	Importance
CORE	16_30	NRRPQDVKFPGGGQI	0.023
	21_35	DVKFPGGGQIVGGVY	0.074
	26_40	GGGQIVGGVYLLPRR	0.165
	31_45	VGGVYLLPRRGPRLG	0.118
	36_50	LLPRRGPRLGVRATR	0.058
	41_55	GPRLGVRATRKTSER	0.015
	61_75	RRQPIPKARQPEGRA	0.020
	66_80	PKARQPEGRAWAQPG	0.013
	76_90	WAQPGYPWPLYGNEG	0.054
	101_115	RGSRPSWGPTDPRRR	0.013
E2	1_20	ETHVTGGNAGRTTAGLVGLL	0.064
	76_95	CRRLTDFAQGWGPISYANGS	0.005
	86_105	WGPISYANGSGLDERPYCWH	0.015
	121_140	GPVYCFTPSPVVVGTTDRSG	0.007
	131_150	VVVGTTDRSGAPTYSWGAND	0.029
	181_200	CGAPPCVIGGVGNNTLLCPT	0.002
	191_210	VGNNTLLCPTDCFRKHPEAT	0.010
	201_220	DCFRKHPEATYSRCGSGPWI	0.014
	216_235	SGPWITPRCMVDYPYRLWHY	0.007
	226_245	VDYPYRLWHYPCTINYTIFK	0.007
NS5	121_170	VTRVGDFHYVTGMTTDNVKCPCQVPAPEFFTEVDGVRLHRYAPACKPLLR	0.014
	261_310	WRQEMGGNITRVESENKVVILDSFEPLQAEEDEREVSVPAEILRRSRKFP	0.045
	271_320	RVESENKVVILDSFEPLQAEEDEREVSVPAEILRRSRKFPRAMPIWARPD	0.035
	281_330	LDSFEPLQAEEDEREVSVPAEILRRSRKFPRAMPIWARPDYNPPLLESWK	0.068
	291_340	EDEREVSVPAEILRRSRKFPRAMPIWARPDYNPPLLESWKDPDYVPPVVH	0.019
	311_360	RAMPIWARPDYNPPLLESWKDPDYVPPVVHGCPLPPAKAPPIPPPRRKRT	0.046
	371_420	ALAELATKTFGSSESSAVDSGTATASPDQPSDDGDAGSDVESYSSMPPLE	0.008
	381_430	GSSESSAVDSGTATASPDQPSDDGDAGSDVESYSSMPPLEGEPGDPDLSD	0.012
	391_440	GTATASPDQPSDDGDAGSDVESYSSMPPLEGEPGDPDLSDGSWSTVSEEA	0.016
NS3	29_96	QVEGEVQVVSTATQSFLATCVNGVCWTVYHGAGSKTLAGPKGPITQMYTNVDQDLVGWQAPPGARSLT	0.008
	81_130	QDLVGWQAPPGARSLTPCTCGSSDLYLVTRHADVIPVRRRGDSRGSLLSP	0.006
	391_440	AYYRGLDVSVIPTSGDVIVVATDALMTGFTGDFDSVIDCNTCVTQTVDFS	0.011

## Discussion

Immuno-diagnosis of infectious disease is based on the ability to detect the presence of disease-related antibodies in clinical samples, such as serum. For this, Mario Geysen produced pathogen-defining “Pepscans” comprised of tiled overlapping synthetic-peptides that served as bait in solid phase immunoassays (15). Here, as an alternative to synthetic-peptides, we employ comprehensive phage-displayed arrays of deconvoluted viral antigens, producing “Domain-Scans”. The use of phage-display, as a means to present peptides for antibody interrogation, offers a number of advantages: (1) Peptides ranging from tens to hundreds of amino acids can easily be expressed; (2) Once cloned, the peptide library can be amplified to produce high titer-stock solutions that can be replenished endlessly; (3) The screening of phage-display peptide arrays can be conducted in small manageable micro-volumes, making phage-displayed Domain-Scans particularly amenable to high-throughput applications, screening numerous samples easily; (4) NGS multiplexing allows the analysis of the affinity-selected peptides, generating mega-data portraying a broader and more comprehensive view of the humoral immune response to infection and other morbidities.

Such application of NGS and T7 phage-display was reported previously by Xu and colleagues in their production of “VirScans” (16). The VirScan library contains a total of 93,904 56-mer peptides that correspond to over 1,000 strains of 206 human infectious viruses. Combined with NGS, Xu and colleagues have been able to study the diversity of viruses that infect human populations, survey the range that different viruses infect individuals and identify antigenic epitopes of diagnostic value (16). The application of the VirScans has already proven useful (17–19).

In this study, we created six phage-displayed Domain-Scans that represent two pathogens, HIV-1 and HCV, containing a total of 339 peptides of 15, 20, and 50 amino acids in length. These Domain-Scans were screened in two multiplex assays (using either Protein VIII or Protein III) against serum samples taken from HIV-1 positive individuals, HCV positive individuals and compared against otherwise healthy individuals. The objective of these analyses was to test the diagnostic performance of our multiplex platform and to identify specific peptide domains that best represent the humoral response raised against either HIV-1 or HCV infection. These domains were used collectively as bait in mixed reactions to measure the presence of specific and corresponding antibodies, independent of one another. The rationale underlying this combinatorial approach (8) is that measuring antibody binding to multiple markers individually, enhances diagnostic power. Demonstrating antibody binding to distant and discontinuous segments of a viral antigen indicates multiple and independent B-cell encounters with the pathogen. Consequently, confidence in a correct diagnostic call increases with the ability to confirm multi-B-cell reactions, even within a given antigen.

Our results illustrate that for the separate viral antigens we detected several specific domains that were found to be effective diagnostic markers, listed in **Tables 3** and **4**. Notably, overlapping domains often cluster indicating strong particularly immunogenic regions within a single antigen. Thus, for example in the NS5 antigen of HCV there were three well defined regions: (i) 121-170, (ii) 261-360, and (iii) 371-440 (**Figure 6**). These separate clusters would indicate that at least three different B-cell clones and possibly more produce anti-NS5 antibodies. Similarly, the situation in HIV-1 gp160 would indicate that at least 6 different B-cell events generate antibodies against the HIV-1 envelope (**Figure 6** and detailed in **Table 3**). The region of eight overlapping 50-mer peptides, from residue 521 to 640, most likely represents at least three distinct, and probably more, epitopes. Thus, for example, the complete well-known dominant pentameric loop of gp41 (residues 590-CSGKLIC-596 (20–22),) appears in three of the eleven affinity selected 50-mer domains. Interestingly, domain 591-640 contains the sequence SGKLIC, missing the first cysteine residue of the disulfide loop. We have previously reported that HIV-1 infected individuals can discriminate both the looped and linear forms of this epitope of gp41 (21), which is supported by the fact that the Domain 591-640 was selected, although relatively poorly. The previously known “573-LAVERY-578” epitope (23–25) is represented in Domain 541-590, independent of the pentameric loop, indicating that this domain has distinct epitopes as well. Finally, the Domains 271-340 represent a second cluster of three overlapping domains and does not contain either the LAVERY epitope or the pentameric loop and thus, must be recognized by yet another B-cell clone. Use of shorter peptide Domains increases the resolution of the assay as is illustrated in the response to the HCV CORE antigen. Multiple independent B-cell clones generate antibodies against this antigen as is illustrated by the two extended clusters of Domains selected by HCV positive sera. As Domain 16-30, which initiates the first cluster, does not overlap at all with the last peptide, residues 41-55, one can conclude that binding to the region 16-55 reflects at least two independent B-cell events. Similarly, one can conclude that the second cluster in the CORE, residues 61-90 binds antibodies derived from at least two independent clones as well. Thus, greater credence and robustness in diagnosis is gained with the ability to detect multiple B-cell responses to a given antigen, a measure that would be lost when using antigen mixtures as in ELISA tests.

Further improvement of combinatorial diagnostics is gained through the implementation of the supervised machine-learning approach illustrated in this study. According to this approach, the weights of the different domains were computed in the training phase of the algorithm, from known cases (HIV-1 and HCV compared to the healthy individuals). The benefit of the machine-learning approach is that it can improve when more data are analyzed: As the number of known cases increases, it is expected that better weighting of the different domains is achieved, leading to better classification accuracy. Another advantage of the machine-learning approach developed here is that the classifier can learn inter-relationships between the separate domains. For example, if each of five separate domains is marginally enriched and not in itself statistically significant, the trained classifier can learn that such a combined signal is enough to classify an unknown sample as positive. The trained classifier can also learn that an enrichment of five other domains may not be enough to classify an unknown sample as positive. Thus, our trained classifier does not merely count the number of statistically significant domains, but rather, specifically considers which domains are enriched, and to which extent. Similarly, the classifier can detect “sticky” domains, which are highly enriched, yet are non-informative for classification. Moreover, the machine-learning classifier does not only provide a binary decision regarding whether a sample is positive or not for a specific pathogen, but rather, it provides a score reflecting how confident a classification is. This allows pointing to samples for which a repeated test should be performed or should be tested by complementary methods.

The application of disease-defining phage-displayed diagnostic markers could be especially important in screening blood donations for multiple pathogen contaminations and thus improve transfusion safety. It is anticipated that by using the Domain-Scan approach and supervised combinatorial diagnostics it should be possible to construct effective epitope arrays for dozens to even hundreds of specific pathogens of concern that could be screened in a single multiplex assay to secure safe blood donations and disqualify those suspected of pathogen contamination.

## Data Availability Statement

The original contributions presented in the study are included in the article/**Supplementary Material**. Further inquiries can be directed to the corresponding authors.

## Ethics Statement

Ethical review and approval was not required for the study on human participants in accordance with the local legislation and institutional requirements. The patients/participants provided their written informed consent to participate in this study.

## Author Contributions

SH-N and YW-O - design and construction of libraries and execution of experiments. NW, OA, and TP - computational pipeline design and execution. NW, OA, HA, and TP - data preparation. YM - HCV biology and epidemiology. ES - HCV biology. DS - HIV serology. SH-N, YWO, NW, TP, and JMG - wrote the primary draft. SH-N completed the manuscript writing with the contributions from all authors. TP and JMG supervised the study. SH-N, YW-O, and NW equally contributed. All authors contributed to the article and approved the submitted version.

## Funding

This research was supported by two Grants of the Israel Science Foundation to JG. NW, OA, and HA were supported in part by a fellowship from the Edmond J. Safra Center for Bioinformatics at Tel Aviv University. NW was supported in part by the Manna Center Program for Food Safety and Security at Tel Aviv University. OA was supported in part by the Dalia and Eli Hurvitz Foundation. HA was supported in part by Humboldt Research Fellowship for Postdoctoral Researchers from the Alexander von Humboldt Foundation. YM and ES were supported in part by a grant from the Dr. Sima Lior research. SH-N and YW-O were recipients of Jakov, Miriana, and Jorge Saia Fellowships.

## Conflict of Interest

The authors declare that the research was conducted in the absence of any commercial or financial relationships that could be construed as a potential conflict of interest.
